# Investigation of the Match Relation between Steel Fiber and High-Strength Concrete Matrix in Reactive Powder Concrete

**DOI:** 10.3390/ma12111751

**Published:** 2019-05-29

**Authors:** Guangyao Yang, Jiangxiong Wei, Qijun Yu, Haoliang Huang, Fangxian Li

**Affiliations:** School of Materials Science and Engineering, South China University of Technology, Guangzhou 510640, China; yangguangyao1988@outlook.com (G.Y.); concyuq@scut.edu.cn (Q.Y.); h.l.huang@msn.com (H.H.); msfxli@scut.edu.cn (F.L.)

**Keywords:** match, steel fiber, reactive powder concrete, strength, flexural toughness

## Abstract

This study investigated the strength and toughness of reactive powder concrete (RPC) made with various steel fiber lengths and concrete strengths. The results indicated that among RPC samples with strength of 150 MPa, RPC reinforced with long steel fibers had the highest compressive strength, peak strength, and toughness. Among the RPC samples with strength of 270 MPa, RPC reinforced with short steel fibers had the highest compressive strength, and peak strength, while RPC reinforced with medium-length steel fibers had the highest toughness. As a result of the higher bond adhesion between fibers and ultra-high-strength RPC matrix, long steel fibers were more effective for the reinforcement of RPC with strength of 150 MPa, while short steel fibers were more effective for the reinforcement of RPC with strength of 270 MPa.

## 1. Introduction

Reactive powder concrete (RPC) is an advanced cement-based material [1,2,3,4]. It has been reported to have remarkable mechanical performance, such as compressive strength between 200 and 800 MPa, flexural strength between 30 and 60 MPa, fracture energy between 1200 and 40,000 J/m^2^, Young’s modulus between 50,000 and 60,000 MPa, and ultimate tensile strain in the order of 1% [5]. Its low permeability, dense microstructure, and superior mechanical properties make RPC a high-performance concrete. This is generally achieved by a microstructural engineering approach, including the utilization of admixtures, reduction of the water-to-cementitious material ratio, very fine particle size, and reduction of the CaO-to-SiO_2_ ratio by introducing silica components and excluding coarse aggregates [6,7,8]. Due to its outstanding performance, RPC has shown great potential in a wide variety of applications, such as civil engineering structures, impact-resistant structures, nuclear engineering structures, and corrosion-resistant structures [9,10].

However, RPC is still a quasi-brittle material. The inclusion of steel fiber can significantly enhance the toughness of RPC and overcome its disadvantage of high brittleness. Steel fibers play a key role in decreasing crack initiation, controlling crack propagation, and effectively increasing the compressive, tensile, and flexural strength [11,12,13,14,15]. 

The mechanical performance of steel fiber-reinforced RPC is affected by many factors. Al-Tikrite [16] investigated the effects of steel fiber type, content, and geometry on the mechanical properties of RPC. It was found that the addition of 4% industrial micro-steel fiber achieved the highest increase in compressive strength and tensile strength. The toughness was increased by increasing the volume content of steel fibers. The addition of 4% industrial micro-steel fiber and 3% waste steel fiber individually achieved an increase in the typical toughness of the RPC by 249.8% and 158.8%, respectively. Wu [17] reported that steel fiber content and shape had significant effects on the compressive and flexural behavior of RPC. Steel fiber content had limited effect on first-crack strength and first-crack deflection but showed considerable effects on the peak load of the flexural load–deflection curve. Tomasz [18] observed that both the volume of steel fibers and the curing conditions influence the flexural behavior of RPC. Mostofinejad [19] studied the effects of different mixtures and cure treatments to determine the optimum parameters for the enhancement of RPC compressive strength. 

Among all the aforementioned factors influencing RPC, steel fiber length has also received attention from many researchers. Olivito [20] reported that the length of steel fibers influences the post-cracking behavior and the tensile strength, while it has a little effect on the compressive strength. Ipek [21] stated that steel fiber size and pre-setting pressure influence the flexural strength and fracture toughness of RPC. Abbas [22] investigated the influence of steel fiber length and content on RPC mechanical and durability performance. The results showed that RPC mixtures incorporating short steel fibers exhibited enhanced flexural properties compared to mixtures with a similar volume of longer steel fibers. Sovjak [23,24] found that the effective fracture energy of ultra-high-performance fiber-reinforced concrete was dependent on both the fiber volume fraction and the fiber aspect ratio, and the fracture energy increased as the aspect ratio increased. Xia [25] studied the effects of steel fiber length on the mechanical properties of RPC and found that steel fiber length had a small impact on the compressive strength, but that the fracture energy sharply decreased by about 50% when the steel fiber length was reduced from 12 to 3 mm. Kim [26] reported that the mechanical properties of a concrete mixture using a single steel fiber length of 13 mm were better than those of mixtures with fiber lengths of 16.5 or 19.5 mm. 

The previous studies discussed above gave contradictory conclusions about the relationship between steel fiber length and reinforcement effect on RPC. This could be caused by differences in the properties of concrete, which would obscure the relation between steel fiber and RPC matrix. Currently, little information is available about the relation between steel fibers of different lengths and mechanical properties of RPC matrices. Accordingly, this study experimentally investigated the mechanical properties, including compressive strength and flexural toughness, of three RPC reinforced with different lengths of steel fibers and exhibiting different strengths. Then, the match relation between fiber length and reinforcement effect on the various RPCs was analyzed on the basis of the bond adhesion between the fibers and the matrix.

## 2. Materials and Methods

### 2.1. Raw Materials

Ordinary Type II, 52.5-grade Portland cement (Zhujiang Cement Co. Ltd., Guangzhou, China) and silica fume (Elkem Co.Ltd., Shanghai, China) with the chemical composition examined by x-ray fluorescence shown in Table 1 were used in this work. The sand used was sieved local natural sand with maximum particle size of 315 μm. The mean particle size of fine quartz powder used was 10 μm. A polycarboxylate-based superplasticizer with water-reducing efficiency greater than 30% was chosen for the preparation of RPC. The properties of the steel fibers used in the experiment are shown in Table 2.

### 2.2. Mixture of Concrete Matrix and Its Compressive Strength

Table 3 summarizes the control mixture proportions of RPC with different components used in the testing program. We used RPCs with steel fiber contents of 0.5% (S-RPC: 0.5% short steel fiber-reinforced RPC, M-RPC: 0.5% medium-length steel fiber-reinforced RPC, and L-RPC: 0.5% long steel fiber-reinforced RPC) and 2.0% (SS-RPC: 2.0% short steel fiber-reinforced RPC, MM-RPC: 2.0% medium-length steel fiber-reinforced RPC, and LL-RPC: 2.0% long steel fiber-reinforced RPC) by volume. The superplasticizer dosage was fixed at 3.0% by mass of the cementitious materials.

### 2.3. Sample Preparation and Experiments

Dry powders, including cement, silica fume, natural sand, and quartz powder, were first mixed for 3 min. This was followed by the addition of water and superplasticizer and mixing for 6 min. Afterwards, fibers were gradually added by hand and mixed for a minimum of 6 min until the ingredients were uniformly distributed. When the mixtures were ready, they were cast into oiled molds and vibrated for consolidation. The specimens were demolded after 1 day and then placed in 90 °C water for 7 days of curing. For 24 h prior to the tests, the specimens were allowed to air-dry in the laboratory. For each mixture, three cubes measuring 70.7 × 70.7 × 70.7 mm^3^ and six beams measuring 40 × 40 × 160 mm^3^ were prepared. After drying, the beams were slotted as described below, and all specimens were wiped to remove debris.

Compressive strength tests were carried out on the cube specimens. Three-point flexural testing with displacement control was conducted on the beam specimens (universal material testing machine, Suns Co. Ltd., Shenzhen, China, 0.05 mm/min control speed, 150 mm span). Before testing, the beam specimens were cut with a slot 12 mm deep. Each value presented in the results is the average of three specimens. A total of 144 specimens were tested in this study.

During the flexural tests, the load and the mid-span deflection were recorded by a computer, which generated and printed load–displacement curves. The first-crack strength and flexural toughness of the load–displacement curves were evaluated according to ASTM C78 and C1018 [27,28]. The toughness index I_20_ is the value determined by dividing the area up to a deflection of 10.5 times the first crack deflection by the area up to the first crack. It reflects the ability of concrete to absorb energy after cracking. In general, the higher the toughness index is, the greater the energy absorption, which means greater toughness. 

**Figure 1:**
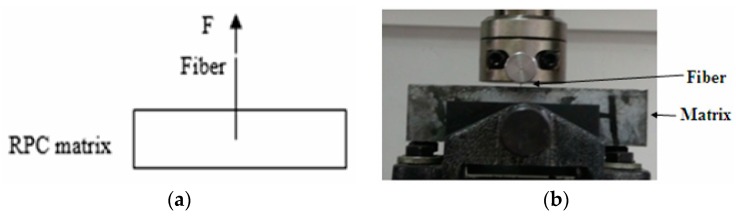
Single-fiber pull-out test: (**a**) schematic and (**b**) photo.

In addition, single-fiber pull-out tests [29,30] were conducted to measure the interfacial adhesion property between steel fibers and the RPC matrix. The fibers were embedded to depths of 5.0, 7.5, or 10.0 mm in the middle of RPC beams during their preparation. The final dimension of a single-fiber pull-out sample is shown in Figure 1. Pull-out tests were conducted on specimens with a universal material testing machine at a speed of 0.1 mm/min. Three tests were conducted on each sample to obtain the average maximum load. A total of 27 specimens were subjected to pull-out tests.

The average bond strength of the fiber–matrix interface was computed by dividing the average maximum pull-out load by the bonding area cross section:

(1) $$\tau =\frac{F}{\pi \mathrm{ds}}$$

where τ is the bond strength of the fiber–matrix interface, F is the maximum pull-out load, d is the fiber diameter, and s is the embedded length of the fiber.

## 3. Mechanical Property Test Results

### 3.1. Compressive Strength

**Figure 2:**
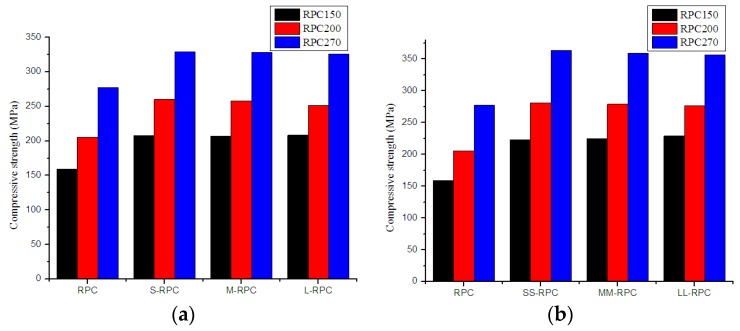
Effect of steel fiber length on the compressive strength of RPCs: (**a**) 0.5% fiber content and (**b**) 2.0% fiber content.

As can be seen from Figure 2, all steel fibers generally greatly increased the compressive strength of all RPC batches, and the strength increased with increasing fiber content. Steel fiber length had a small effect on the compressive strength of RPC. To be specific, long steel fibers showed slightly better strength improvement for RPC150, while short steel fibers showed slightly better strength improvement for RPC200 and RPC270.

### 3.2. Flexural Load–Deflection Curves

**Figure 3:**
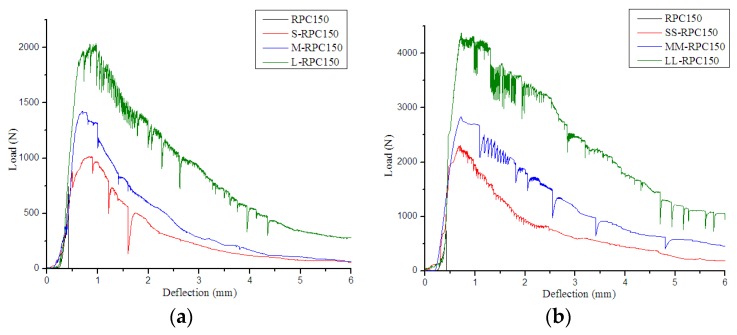
Flexural load–deflection curves for RPC150: (**a**) 0.5% fiber content and (**b**) 2.0% fiber content.

**Figure 4:**
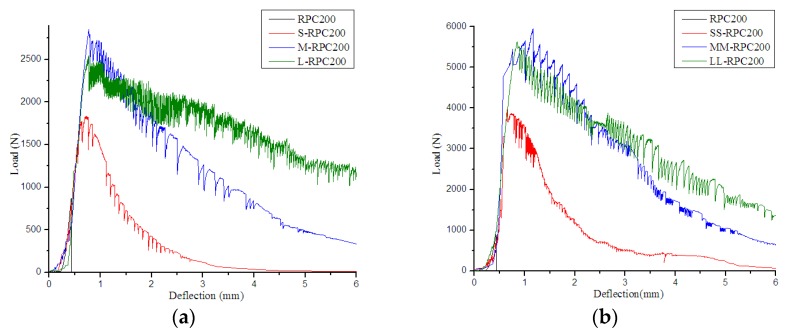
Flexural load–deflection curves for RPC200: (**a**) 0.5% fiber content and (**b**) 2.0% fiber content.

**Figure 5:**
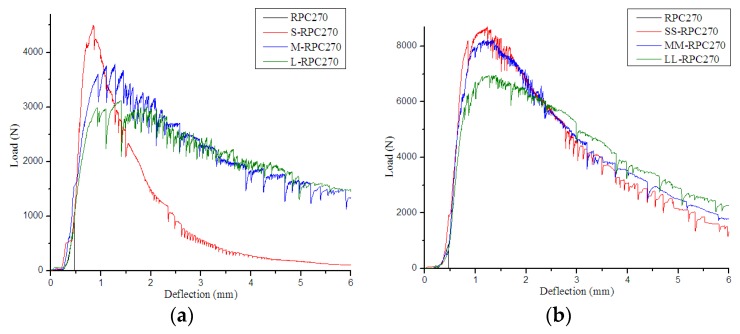
Flexural load–deflection curves for RPC270: (**a**) 0.5% fiber content and (**b**) 2.0% fiber content.

Figure 3, Figure 4 and Figure 5 compare the flexural load–deflection curves of RPC beams of various strengths reinforced with steel fibers of different lengths. It was found that the unreinforced RPC demonstrated relatively brittle behavior. The behavior of the samples during the flexural test was almost linear elastic up to the peak-load values, but then the curves sloped downward until the complete separation of the samples into two parts. However, when steel fibers were incorporated, the samples could sustain further load after cracking. The decreasing deflection trends were flatter, and the flexural curves depended on the properties of the added fibers and their combinations. In addition, with increased steel fiber content, the areas under the curve increased, with greater peak load. Moreover, with increased steel fiber length, the curves became more voluminous in all batches, with higher peak load in RPC150 but lower peak load in RPC270.

**Figure 6:**
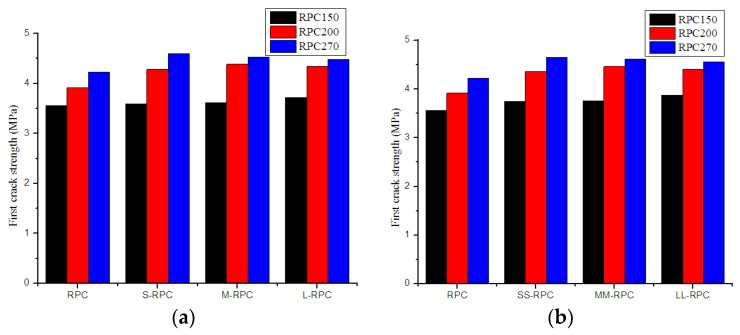
Effects of steel fiber length on first-crack strength of RPC: (**a**) 0.5% fiber content and (**b**) 2.0% fiber content.

**Figure 7:**
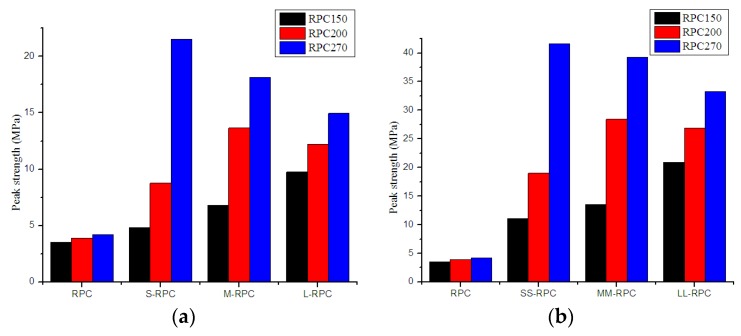
Effects of steel fiber length on peak strength of RPC: (**a**) 0.5% fiber content and (**b**) 2.0% fiber content.

**Figure 8:**
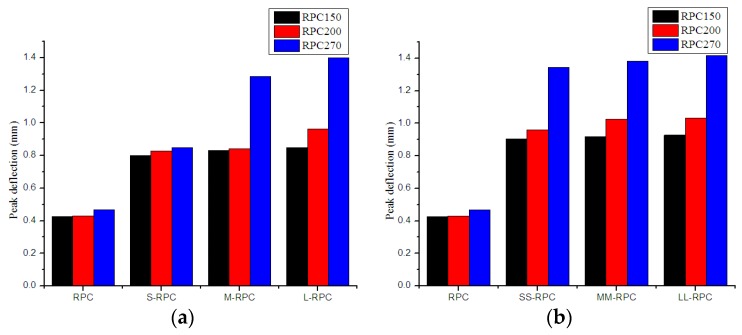
Effects of steel fiber length on peak deflection of RPC: (**a**) 0.5% fiber content and (**b**) 2.0% fiber content.

The effects of steel fiber length on first-crack strength, peak strength, and peak deflection of RPC samples with different strengths are illustrated in Figure 6, Figure 7 and Figure 8. It is obvious that, with the reinforcement of steel fibers, the first-crack strength, peak strength, and peak deflection all increased significantly, and they increased with increasing fiber content. From the figures, RPC150 with long steel fibers had the highest first-crack strength, peak strength, and peak deflection. RPC200 and RPC270 with long steel fibers also had the highest peak deflection (RPC200 with medium-length steel fibers had the highest peak deflection at 2% fiber content), but RPC200 with medium-length steel fibers had the highest first-crack strength and peak strength, while RPC270 with short steel fibers had the highest first-crack strength and peak strength.

For RPC150, the first-crack strength increased by 1.1% and 5.3% with 0.5% and 2.0% short steel fiber and increased by 4.7% and 9.1% with 0.5% and 2.0% long steel fiber, respectively. The peak strength increased by 36.3% and 211.1% with 0.5% and 2.0% short steel fiber and increased by 174.2% and 489.1% with 0.5% and 2.0% long steel fiber, respectively. For RPC270, the first-crack strength increased by 8.7% and 10.0% with 0.5% and 2.0% short steel fiber and increased by 6.0% and 7.7% with 0.5% and 2.0% long steel fiber, respectively. The peak strength increased by 409.1% and 884.8% with 0.5% and 2% short steel fiber and increased by 253.4% and 687.6% with 0.5% and 2.0% long steel fiber, respectively. In general, the first-crack strength and peak strength of RPC150 increased with increasing fiber length. In comparison, these two strengths of RPC270 decreased with increasing fiber length.

### 3.3. Flexural Toughness Index

**Figure 9:**
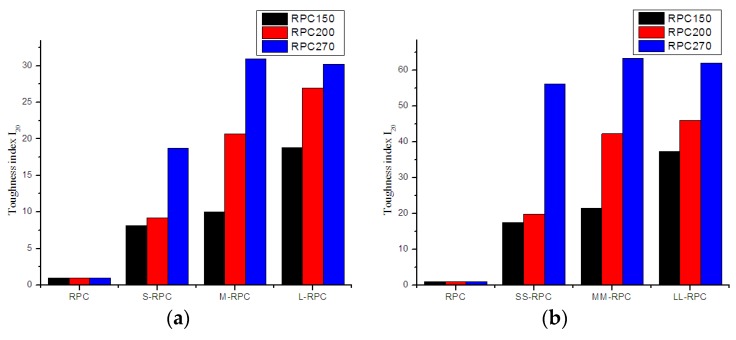
Effects of steel fiber length on the toughness index I_20_ of RPC: (**a**) 0.5% fiber content and (**b**) 2.0% fiber content.

The effects of steel fiber length on the flexural toughness index I_20_ of RPCs with different strengths are summarized in Figure 9. The data indicated that the toughness index of all RPC batches was increased greatly with all steel fibers, and the index increased with increasing fiber content. As shown in RPC150 and RPC200, short steel fibers gave the lowest toughness index, and long steel fibers gave the highest toughness index. For RPC270, short steel fibers still gave the lowest toughness index, and medium-length steel fibers gave the highest toughness index. 

From the figure, the toughness index increased with increasing fiber length in RPC150. The toughness index with 0.5% content of short, medium, and long steel fibers was 8.17, 10.02, and 18.82 times higher, respectively, at the first crack. The toughness index with 2.0% content of short, medium, and long steel fibers was 17.51, 21.48, and 37.35 times higher, respectively, at the first crack. However, for RPC270, the toughness index increased in the order of short, long, and medium steel fibers. The increase was 18.7, 30.2, and 31.0, and 56.06, 61.99, and 63.31 times with 0.5% and 2.0% fiber content, respectively. To summarize, RPC150 and RPC200 reinforced with long steel fibers showed better flexural toughness, while RPC270 reinforced with medium-length steel fibers showed better flexural toughness.

## 4. Discussion

### 4.1. Mechanism of Fiber Reinforcement

The destruction process of concrete structures comprises the initiation, growth, and coalesces of microcracks, until macrocracks appear and finally produce failure. The main function of fiber reinforcement is to control the cracking and prevent catastrophic failures in fiber-reinforced cement-based material. 

In steel fiber-reinforced RPC, at the pre-cracking stage, the RPC matrix transfers a load to the steel fibers through interfacial adhesion under external force. Both steel fibers and RPC matrix sustain the load together. The load-carrying capacity was improved, and cracking was delayed compared to unreinforced RPC. After cracking, only the steel fibers sustained the load through mechanical interlock and friction at the fiber–matrix interface. As the fibers were pulled out, the crack energy was absorbed, and the deflection and load increased. Thus, the interfacial bond adhesion property between the steel fibers and the RPC matrix plays an important role that influences the enhancement effect of fibers and final mechanical properties of modified RPCs.

Generally, the RPC samples with steel fibers exhibited a much more ductile behavior. It can be seen from the test results that all lengths of steel fibers greatly increased the strength, deflection, and toughness.

### 4.2. Single-Fiber Pull-Out Test

**Figure 10:**
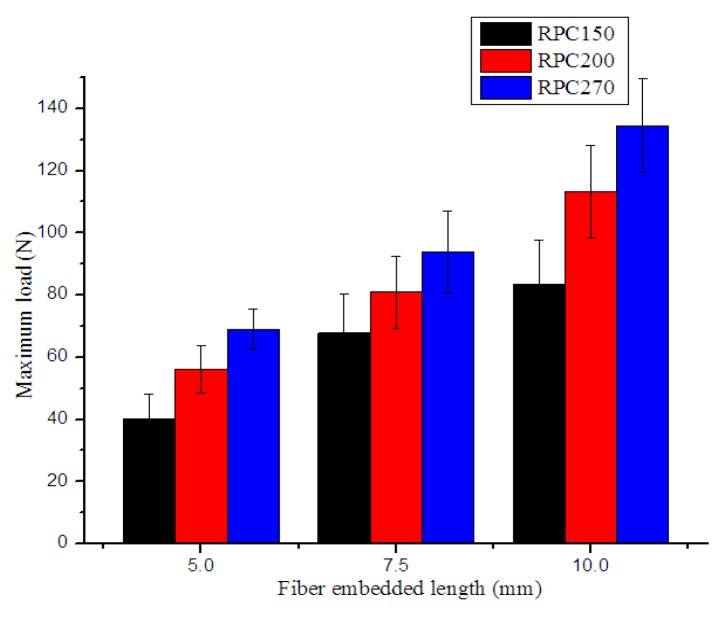
Effects of embedded fiber length on maximum load.

**Figure 11:**
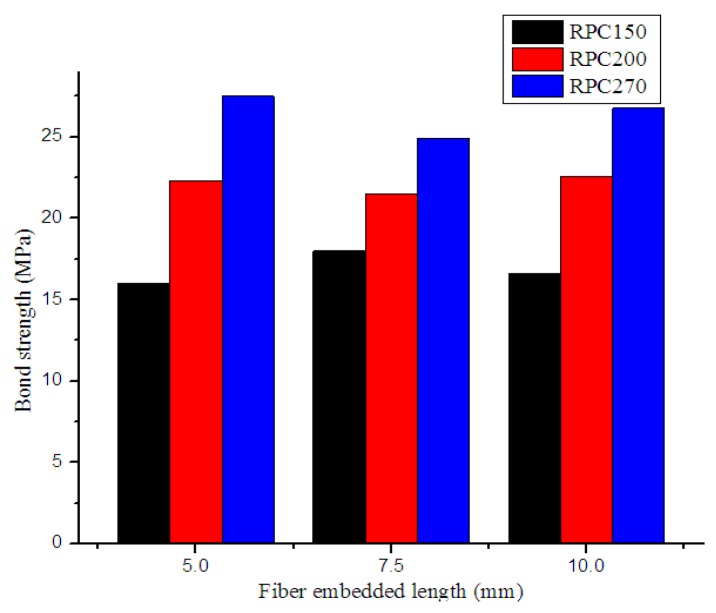
Effects of embedded fiber length on bond strength.

To obtain more detailed information about the reinforcement of RPCs by steel fibers, pull-out tests of steel fibers of different lengths embedded in three different RPC matrices were conducted. The test results are presented in Figure 10 and Figure 11. The results indicated that maximum pull-out load increased with increasing embedded fiber length or matrix strength, while bond strength only increased with increasing matrix strength. This is because with increased RPC matrix strength, the RPC became denser and more compact. Thus, the bond adhesion between steel fibers and the RPC matrix was also enhanced. 

The pull-out test results are similar to those of previous researches. Beglarigale [31] found that the fiber–matrix bond characteristics (pull-out peak load and debonding toughness) improved as the embedment length of the fiber increased, and dense microstructure led to better bond characteristics between steel fiber and RPC matrix. Lebdeh [32] stated that the maximum pull-out load and total pull-out energy increased as matrix strength increased for the fibers that did not rupture, and some fibers ruptured when pulled out from very high strength concrete matrix. Alberti [33] reported that the critical fiber length, as well as the overall pull-out behavior, were strongly influenced by the type of matrix in which the fiber was embedded, and embedded length longer than critical length led to fiber rupture.

### 4.3. Match Relation between Steel Fibers and RPC Matrix

As can be seen from the test results, RPC150 reinforced with long steel fibers had the highest compressive strength, first-crack strength, peak strength, peak deflection, and toughness index. In comparison, RPC270 reinforced with short steel fibers had the highest compressive strength, first-crack strength, and peak strength, and RPC270 reinforced with medium-length steel fibers showed the highest toughness index, while RPC270 reinforced with long steel fibers only showed the highest peak deflection. In summary, the use of long steel fibers is more effective for the reinforcement of RPC150, while short steel fibers are more effective for the reinforcement of RPC270.

**Figure 12:**
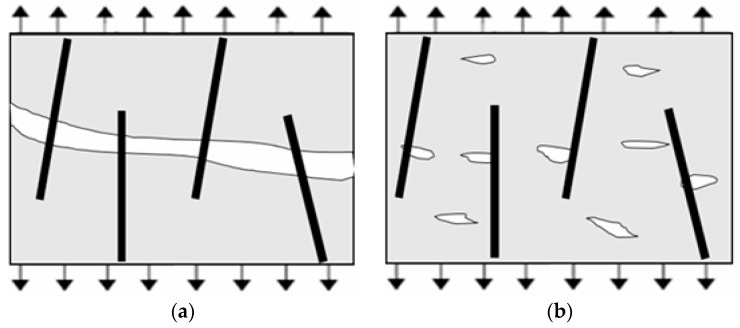
Mechanism of long-fiber reinforcement: (**a**) concrete with macrocracks and (**b**) concrete with microcracks.

Long fibers showed a better improvement effect for RPC150 due to increased slowing of the propagation of macrocracks because of an enhanced bridge effect for large cracks by long fibers, as illustrated in Figure 12. Hence, the flexural load–deflection curves showed a flatter descent and larger deflection at the post-cracking stage. In addition, in the descending part of the curves, a jagged shape was more evident in the samples with longer fibers, which indicated that steel fibers were gradually pulled out from the matrix. Additionally, as illustrated from the single-fiber pull-out test, although increased fiber length produced small changes in bond strength between fibers and RPC matrix, the maximum pull-out load and total pull-out energy clearly increased, which was also more conducive to abate or inhibit the growth of cracks. 

**Figure 13:**
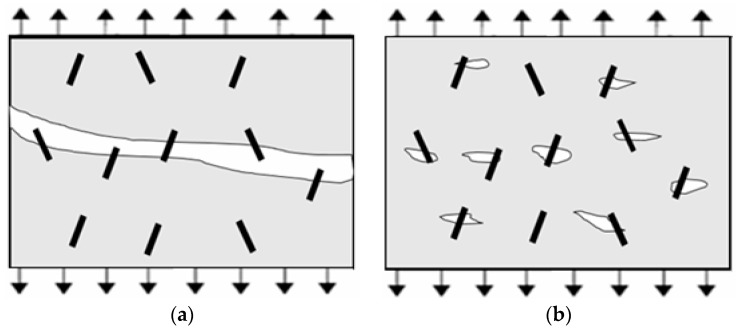
Mechanism of short-fiber reinforcement: (**a**) concrete with macrocracks and (**b**) concrete with microcracks.

However, in ultra-high-strength RPC, for example RPC270, as shown in Figure 13, because of the use of a low water-to-cementitious material ratio, very fine powder, compact mix proportion, internal interface, porosity, and flaws became much smaller but increased in number. Therefore, at the same volume fraction, more widely dispersed short fibers could reduce the stress concentration at these flaws, which delayed the initiation and growth of microcracks at the pre-cracking stage and produced higher first-crack strength and peak strength. Moreover, the interfacial zone brought by fibers [34] between short fibers and the RPC matrix was shorter than that for long fibers, which would produce smaller flaws than those produced by long fibers. More importantly, increasing the strength of the RPC enhanced the bond adhesion between fibers and matrix. Thus, the improvement effect of short fibers also increased, while long fibers were more likely to rupture. This caused short fibers to effectively arrest cracks at both the pre-cracking and the post-cracking stage. For these reasons, short fibers produced better improvements in RPC270.

## 5. Conclusions

In this study, the compressive strength and flexural toughness of RPCs reinforced with different lengths of steel fibers, exhibiting three different strengths, were experimentally investigated. Then, the relation between the fiber length and the reinforcement effect on RPC of various strengths was analyzed on the basis of the bond adhesion between fibers and matrix. The following conclusions can be drawn, based on the results and discussion:

(1) For RPC150, the samples reinforced with long steel fibers had the highest compressive strength, first-crack strength, peak strength, and toughness index.
(2) For RPC270, the samples reinforced with short steel fibers had the highest compressive strength, first-crack strength, and peak strength, while the toughness index of RPC270 reinforced with medium-length steel fibers was the highest.
(3) Consequently, we found a match relation between the length of the steel fibers and the strength of the RPC matrix. As a result of the higher bond adhesion between fibers and ultra-high-strength RPC matrix, long steel fibers were more effective for reinforcing RPC150, while short steel fibers were more effective for reinforcing RPC270.

## Figures and Tables

**Table 1:** Chemical composition of raw materials (wt.%).

Chemical Composition	SiO_2_	Al_2_O_3_	Fe_2_O_3_	CaO	MgO	K_2_O	Na_2_O	SO_3_	TiO_2_
Cement	19.66	4.29	3.37	62.52	0.85	0.62	0.08	2.61	0.24
Silica fume	95.74	0.50	/	1.25	0.63	1.07	0.33	0.15	/

**Table 2:** Properties of steel fibers. S: short, M: medium, L: long.

Abbreviation	Length (mm)	Equivalent Diameter (mm)	Aspect Ratio	Density (g/cm^3^)	Tensile Strength (MPa)	Elastic Module (GPa)	Elongation (%)
S	6	0.16	37.5	7.8	>3000	>210	<4
M	12	0.16	75	7.8	>3000	>210	<4
L	20	0.16	125	7.8	>3000	>210	<4

**Table 3:** Mix proportions of reactive powder concrete (RPC) matrix and its compressive strength.

Batch no.	Cement (C)	Silica Fume (SF/C)	Sand (S/C)	Quartz Powder (Qu/C)	Water (W/C)	Compressive Strength (MPa)
RPC150	1	0.2	1.3	/	0.19	159.24
RPC200	1	0.2	1.3	/	0.15	205.46
RPC270	1	0.25	0.5	0.4	0.17	277.64
